# Differential role of residual metabolic tumor volume in inoperable stage III NSCLC after chemoradiotherapy ± immune checkpoint inhibition

**DOI:** 10.1007/s00259-021-05584-w

**Published:** 2021-10-19

**Authors:** Marcus Unterrainer, Julian Taugner, Lukas Käsmann, Amanda Tufman, Niels Reinmuth, Minglun Li, Lena M. Mittlmeier, Peter Bartenstein, Wolfgang G. Kunz, Jens Ricke, Claus Belka, Chukwuka Eze, Farkhad Manapov

**Affiliations:** 1 Department of Radiology, University Hospital, LMU Munich, Marchioninistr. 15, 81377 Munich, Germany; 2 Department of Radiotherapy and Radiation Oncology, University Hospital, LMU Munich, Munich, Germany; 3 Member of the German Center for Lung Research (DZL), Comprehensive Pneumology Center Munich (CPC-M), Munich, Germany; 4 German Cancer Consortium (DKTK), Partner Site Munich, Munich, Germany; 5 Department of Internal Medicine V, LMU Munich, Munich, Germany; 6 Asklepios Lung Clinic, Munich-Gauting, Germany; 7 Department of Nuclear Medicine, University Hospital, LMU Munich, Munich, Germany

**Keywords:** NSCLC, Immunotherapy, Metabolic tumor volume, Durvalumab, Nivolumab

## Abstract

**Background:**

The PET-derived metabolic tumor volume (MTV) is an independent prognosticator in non-small cell lung cancer (NSCLC) patients. We analyzed the prognostic value of residual MTV (rMTV) after completion of chemoradiotherapy (CRT) in inoperable stage III NSCLC patients with and without immune checkpoint inhibition (ICI).

**Methods:**

Fifty-six inoperable stage III NSCLC patients (16 female, median 65.0 years) underwent ^18^F-FDG PET/CT after completion of standard CRT. rMTV was delineated on ^18^F-FDG PET/CT using a standard threshold (liver SUV_mean_ + 2 × standard deviation). 21/56 patients underwent additional ICI (CRT-IO, 21/56 patients) thereafter. Patients were divided in volumetric subgroups using median split dichotomization (MTV ≤ 4.3 ml vs. > 4.3 ml). rMTV, clinical features, and ICI-application were correlated with clinical outcome parameters (progression-free survival (PFS), local PFS (LPFS), and overall survival (OS).

**Results:**

Overall, median follow-up was 52.0 months. Smaller rMTV was associated with longer median PFS (29.3 vs. 10.5 months, *p* = 0.015), LPFS (49.9 vs. 13.5 months, *p* = 0.001), and OS (63.0 vs. 23.0 months, *p* = 0.003). CRT-IO patients compared to CRT patients showed significantly longer median PFS (29.3 vs. 11.2 months, *p* = 0.034), LPFS (median not reached vs. 14.0 months, *p* = 0.016), and OS (median not reached vs. 25.2 months, *p* = 0.007). In the CRT subgroup, smaller rMTV was associated with longer median PFS (33.5 vs. 8.6 months, *p* = 0.001), LPFS (49.9 vs. 10.1 months, *p* = 0.001), and OS (63.0 vs. 16.3 months, *p* = 0.004). In the CRT-IO subgroup, neither PFS, LPFS, nor OS were associated with MTV (*p* > 0.05 each). The findings were confirmed in subsequent multivariate analyses.

**Conclusion:**

In stage III NSCLC, smaller rMTV is highly associated with superior clinical outcome, especially in patients undergoing CRT without ICI. Patients with CRT-IO show significantly improved outcome compared to CRT patients. Of note, clinical outcome in CRT-IO patients is independent of residual MTV. Hence, even patients with large rMTV might profit from ICI despite extensive tumor load.

## Introduction

Advanced stage III non-small cell lung cancer (NSCLC) represents a heterogenous tumor entity regarding patient and tumor features [1–5] leading to interdisciplinary treatment strategies and regimens in these mostly inoperable patient cohort [6–10]. So far, standard treatment in stage III NSCLC consisted of a combination of platinum-based chemotherapy applied concurrently or sequentially to thoracic irradiation (CRT) leading to improved clinical outcome in terms of local control, metastasis free, and overall survival compared to irradiation alone [11]. Beyond this combined approach, immune checkpoint inhibition (ICI) has evolved as additional treatment option NSCLC patients [12], especially with regard to the first US Food and Drug Administration (FDA) approval for PD-1 inhibition (nivolumab) in 2015 in advanced or metastatic NSCLC [13, 14]. Consequently, further combined treatment regimens with PD-L1 inhibition (pembrolizumab) in the KEYNOTE-189 and 407 trials showed improved clinical outcome in NSCLC patients independent of the PD-L1 status compared to mere standard chemotherapy [15, 16]. Recent ground-breaking clinical data were presented in the PACIFIC trial suggesting a continuous PD-L1 inhibition (Durvalumab) after the completion of standard CRT due to distinctly improved patient outcome considering PD-L1 inhibition after standard CRT as new standard of care [8]. Moreover, additional data suggest more combinatory possibilities to reach long lasting tumor control [12, 17].

PET imaging has gained lasting clinical importance in the therapeutic workup of NSCLC patients [18, 19], e. g., for radiotherapy planning [20], whole body staging [21], or treatment monitoring [22]. Interestingly, the PET-derived metabolic tumor volume (MTV) has evolved as tool for response assessment and prognostication [23, 24]. An association of the residual MTV (rMTV) after completion of CRT with the further disease course has been described, e. g., indicating a cut-off of 25.0 ml rMTV as prognosticator for clinical outcome [25, 26]; moreover, additional data suggested further rMTV cut-offs such as 1.0 ml rMTV [25].

Therefore, we aimed at assessing the prognostic value of the PET-derived rMTV in stage III NSCLC patients after completion of standard CRT with regard to consecutive ICI consolidation (CRT-IO) in direct comparison to stage III NSCLC patients undergoing standard CRT only, to assess whether prognostic stratification using rMTV on PET is also valid for CRT-IO treatment regimens in the light of ICI and changing standards of care.

## Methods

### Patients

Fifty-six patients with histologically proven, inoperable, and locally advanced NSCLC stage IIIA–C (UICC 7th edition) and ^18^F-FDG PET/CT imaging after completion of combined RCT with or without immune checkpoint inhibitor therapy consolidation from clinical routine 2011–2018 were included (during analyses, patients were reclassified according to the 2018 UICC 8th edition). All patients were treated at a single tertiary cancer center. Prior to treatment, basic patient characteristics were assessed. Cranial contrast-enhanced magnetic resonance imaging (MRI) or contrast-enhanced head computed tomography scan (CT) was performed in all cases. All patients received routine blood work to assess kidney function as well as complete blood count and underwent pulmonary function testing. Patients receiving durvalumab maintenance were given durvalumab intravenously at a dose of 10 mg/kg every 2 weeks up to 12 months (24 cycles), until progression or unacceptable toxicity according to the Common Toxicity Criteria for Adverse Events (CTCAE) version 5. Patients receiving nivolumab were treated in the NICOLAS trial [27].

All patients were discussed prior to treatment at the multidisciplinary tumor board, and all patients were deemed inoperable by an experienced group of thoracic surgeons, pulmonologists, and radiation oncologists. Patients with an initial performance status ECOG > 1 and poor lung function (DLCO < 40%, FEV1 < 1 l or on long-term oxygen therapy) were excluded from this analysis.

### Image acquisition and data evaluation

All PET/CT scans were performed at the same institution using a GE Discovery 690 PET/CT scanner (GE Healthcare, Chicago, IL, USA). Scans were initiated 60–90 min after intravenous administration of 20 mg furosemide, 10–20 mg butylscopolamine, and ^18^F-FDG, when no medical contraindication was given. PET/CT examinations were performed in the treatment position (patient’s arms overhead, wingstep) on carbon fiber couch. PET/CT imaging was performed including a diagnostic, contrast-enhanced CT scan in portal-venous phase (350 mg of Imeron at 1.5 ml/kg body weight). PET was acquired with 2.5 min per bed position. Images were reconstructed iteratively using TrueX (three iterations, 21 subsets) with Gaussian post-reconstruction smoothing (2 mm full width at half-maximum). For MTV delineation, a background activity of the liver (mean SUV_liver_) was derived using a 3.0 cm spherical volume of interest (VOI) including the respective standard deviation (SD) within this VOI. The respective cut-off for MTV delineation was set as mean SUV_liver_ + 2 × SD_liver_ [28]. Moreover, the maximal SUV (SUV_max_) of the tumor manifestations was derived. The SUV measurements were performed using automated software in a 3D volume tool (Hybrid Viewer 3D, Hermes Medical Solutions, Stockholm, Sweden).

### Clinical parameters

Beyond the PET-derived MTV, further clinical parameters were assessed including age, sex, histological subtypes, UICC stage (IIIA-C), planning target volume (PTV), reached radiation dose at radiotherapy (RT), application of RCT-IO, and application of previous induction chemotherapy.

### Outcome parameters/tumor progression

Clinical and image-derived parameters were correlated with the patients’ progression-free survival (PFS) as defined by RECIST 1.1 [29–31], local PFS (LPFS), and overall survival (OS) to derive associations of clinical and image-derived parameters with the direct clinical outcome.

### Statistics

IBM® SPSS® Statistics (version 25, IBM Corp., Armonk, NY, USA). Normal distribution was assessed using the Shapiro–Wilk test. Descriptive statistics are displayed as mean + SD or median (range). Kaplan–Meier curves were used for PFS, LPFS, and OS calculation and log-rank test for univariate comparison of PFS, LPFS, and OS regarding ordinary variables; PFS, LPFS, and OS are displayed as median survival with 95% confidence interval (CI). For testing of continuous parameters, a dichotomization was performed either using previously published cut-offs or using median split. Significant parameters from univariate analysis were consecutively included into the multivariate analysis, where results are displayed as hazard ratio (HR) and CI. Correlation analysis was performed using Pearson correlation coefficient. Statistical significance was defined as two-tailed *p*-values < 0.05.

## Results

### Patients/clinical features

Overall, 56 stage III NSCLC patients with a median age of 65 (range, 33–83) years were included (40/56 males (71.4%), 16/56 females (28.6%)). Of those, 9/56 (16.1%) comprised stage IIIA, 27/56 (48.2%) stage IIIB, and 20/56 (35.7%) stage IIIC with an underlying histology of adenocarcinoma in 11/56 (19.7%) cases, squamous cell carcinoma in 41/56 (73.2%), and other entities in 4/56 (7.1%) cases. During CRT, a radiation dose of at least 60 Gray was reached by 47/56 (83.9%) and a dose < 60 Gray in 9/56 (16.1%) patients. 30/56 (53.6%) patients consecutively comprised a planning target volume prior to irradiation of at least 700 ml [32, 33] and 26/56 (46.4%) with a PTV < 700 ml. After completion of CRT, 35/56 (62.5%) patients underwent clinical follow-up (historical data), and 21/56 (37.5%) additionally underwent ICI consolidation being sequential therapy with durvalumab in 12/21 (57.1%) cases and concurrent/sequential ICI with nivolumab in 9/21 cases (42.9%) (please see Table 1).

**Table 1:** Patient characteristics

**Age**	
Median (range)	65 (33–83) yrs
**Sex**	
Male	40 (71.4%)
Female	16 (28.6%)
**Histology**	
Adenocarcinoma	11 (19.7%)
Squamous cell carcinoma	41 (73.2%)
Others	4 (7.1%)
**Stage**	
IIIA	9 (16.1%)
IIIB	27 (48.2%)
IIIC	20 (35.7%)
**Therapy**	
CRT-IO	21 (37.5%)
CRT	35 (62.5%)
**Mode of IO**	
Durvalumab (sequential)	12 (57.1%)
Nivolumab (concurrent/sequential)	9 (42.9%)
**Induction chemotherapy**	
Yes	26 (46.6%)
No	30 (53.6%)
**Cumulative RT dose**	
< 60 Gy	9 (16.1%)
≥ 60 Gy	47 (83.9%)
**Residual MTV**	
Median (range)	4.3 (0.0–144.0) ml
**SUV**_**max**_	
Median (range)	13.8 (2.0–43.0)
**PTV**	
< 700 ml	30 (53.6%)
≥ 700 ml	26 (46.4%)

### rMTV and SUV_max_ on ^18^F-FDG PET/CT

All patients received ^18^F-FDG PET/CT after completion of CRT for rMTV assessment (12 ± 6 weeks, median 10 weeks). Here, a median rMTV of 4.3 (0.0–144.0) ml was observed. No residual activity (i.e., rMTV of 0.0 ml) was present in 18/56 (32.1%) patients, a rMTV ≤ 1.0 ml in 23/56 (41.1%) patients, and a rMTV ≤ 25.0 ml in 41/56 patients (73.2%). Overall, there was a median SUV_max_ of 13.8 (range, 2.0–43.0) (see also Table 1).

### Progression-free survival

Overall, there was a median follow-up time of 52.0 months, and median PFS was 14.2 months. Patients with RCT-IO compared to RCT showed a significantly longer median PFS (29.3 vs. 11.2 months, *p* = 0.034). Also, patients with smaller rMTV (by median split dichotomization using a cut-off of 4.3 ml) comprised a significantly longer PFS than those with larger rMTV (16.3 vs. 11.2 months, *p* = 0.015). Also, SUV_max_ showed a significant association with PFS (29.3 vs. 12.7 months, *p* = 0.049). All other parameters showed no association with PFS (*p* > 0.05). On multivariate analysis, both omission of CRT-ICI (HR 2.6, *p* = 0.009) and larger rMTV > 4.3 ml kept statistical significance (HR 2.4, p = 0.009), whereas SUV_max_ showed no association with PFS on multivariate analysis. All other parameters were not included in the multivariate analysis, respectively (please see Table 2).

**Table 2:** Uni-/multivariate analysis PFS (median overall: 14.2 (11.9–16.5) months)

	Univariate analysis		Multivariate analysis	
Parameter	Median PFS (95% CI)	Significance	Hazard ratio (95% CI)	Significance
**Age**				
< 65 years	15.4 (11.1–19.7)	p = 0.615	-	-
≥ 65 years	14.6 (10.5–18.7)			
**Sex**				
Male	15.3 (11.5–19.1)	*p* = 0.691	-	-
Female	14.2 (7.1–21.3)			
**Histology**				
Adeno	15.4 (7.1–23.7)	*p* = 0.747	-	-
Squamous/other	15.3 (11.0–19.6)			
**Stage**				
IIIA	12.7 (5.7–19.7)	*p* = 0.195	-	-
IIIB	19.7 (5.6–33.8)			
IIIC	11.2 (6.9–15.5)			
**Therapy**				
CRT-IO	29.3 (10.4–48.2)	*p* = 0.034	2.6 (1.3–5.4)	*p* = 0.009
CRT	11.2 (6.1–16.3)			
**Induction chemotherapy**				
Yes	15.4 (13.4–17.4)	*p* = 0.877	-	-
No	12.9 (1.3–24.6)			
**Cum. RT dose**				
< 60 Gy	8.7 (8.4–8.9)	*p* = 0.172	-	-
≥ 60 Gy	16.2 (12.7–19.7)			
**Residual MTV MS**				
< 4.3 ml	29.3 (8.5–50.1)	*p* = 0.015	2.4 (1.2–4.7)	*p* = 0.009
≥ 4.3 ml	10.5 (6.7–14.3)			
**SUV**_**max**_				
< 13.8	29.3 (8.7–49.9)	*p* = 0.049	1.8 (0.9–3.5)	*p* = 0.143
≥ 13.8	12.7 (9.0–16.3)			
**PTV**				
< 700 ml	16.3 (13.5–19.1)	*p* = 0.505	-	-
≥ 700 ml	11.2 (8.2–14.2)			

### Local progression-free survival

In the whole cohort, there was a median LPFS of 20.4 months. Again, patients with RCT-IO compared to RCT showed a significantly longer median LPFS (median not reached vs. 14.0 months, *p* = 0.004). Analogously, patients with smaller rMTV (cut-off of 4.3 ml) showed a significantly longer PFS than those with larger rMTV (49.9 vs. 13.5 months, *p* = 0.001). A larger PTV was also accompanied by a significantly shorter LPFS (34.7 vs. 2.8 months, *p* = 0.017). All other parameters showed no association with clinical outcome in terms of LPFS (*p* > 0.05). On multivariate analysis, both omission of CRT-ICI (HR 3.5, p = 0.004) and larger rMTV > 4.3 ml kept statistical significance (HR 4.2, p < 0.001), whereas PTV showed no significance on multivariate analysis. All other parameters were not included in the multivariate analysis, respectively (please see Table 3).

**Table 3:** Uni-/multivariate analysis LPFS (median overall: 20.4 (3.5–37.3) months)

	Univariate analysis		Multivariate analysis	
Parameter	Median lPFS (95% CI)	Significance	Hazard ratio (95% CI)	Significance
**Age**				
< 65 years	20.4 (12.0–28.8)	*p* = 0.750	-	-
≥ 65 years	15.3 (0.0–43.9)			
**Sex**				
Male	20.4 (8.1–32.7)	*p* = 0.934	-	-
Female	16.9 (0.0–40.9)			
**Histology**				
Adeno	16.3 (6.4–26.2)	*p* = 0.212	-	-
Squamous/other	23.6 (3.4–43.8)			
**Stage**				
IIIA	15.3 (7.7–22.8)	*p* = 0.156	-	-
IIIB	34.7 (18.4–48.6)			
IIIC	14.3 (9.4–19.1)			
**Therapy**				
CRT-IO	Not reached	*p* = 0.004	3.5 (1.5–8.3)	*p* = 0.004
CRT	14.0 (9.3–18.7)			
**Induction chemotherapy**				
Yes	16.9 (7.4–26.4)	*p* = 0.779	-	-
No	23.6 (0–47.3)			
**Cum. RT dose**				
< 60 Gy	10.5 (5.3–15.6)	*p* = 0.059	-	-
≥ 60 Gy	33.8 (15.1–51.9)			
**Residual MTV MS**				
< 4.3 ml	49.9 (5.3–94.5)	*p* = 0.001	4.2 (1.9–9.0)	*p* < 0.001
≥ 4.3 ml	13.5 (7.7–19.3)			
**SUV**_**max**_				
< 13.8	34.7 (6.3–62.9)	*p* = 0.165	-	-
≥ 13.8	15.3 (6.9–23.6)			
**PTV**				
< 700 ml	34.7 (19.5–49.8)	*p* = 0.017	1.9 (0.9–4.0)	*p* = 0.060
≥ 700 ml	12.8 (9.4–16.3)			

### Overall survival

In the whole cohort, the median OS was 52.0 months. CRT-IO was associated with significantly longer OS compared to CRT only (median not reached vs. 25.2 months, *p* = 0.007). Again, rMTV (cut-off 4.3 ml) was associated with significantly longer OS in patients with smaller rMTV (63.0 vs. 23.0 months, *p* = 0.003). A lower SUV_max_ was also associated with OS (63.0 vs. 24.9, *p* = 0.016). PTV also showed significant association with OS, as patients with smaller initial PTV also showed a significantly longer median OS compared to those with larger PTV at CRT initiation (63.0 vs. 23.0, *p* = 0.017). Absolute values of PTV and BTV were not correlated with each other (*r* = 0.219, *p* = 0.105), so that direct inter-correlation effects on multivariate analysis can be excluded. On multivariate analysis, an association with OS was found in the following parameters: CRT HR 3.5, *p* = 0.023; rMTV HR 3.9, *p* = 0.002; and PTV HR 2.6, *p* = 0.026. All other parameters were not associated with OS on univariate analyses including SUV_max_ (*p* > 0.05 each); consecutively, these factors were not included in multivariate analyses (please see Table 4).

**Table 4:** Uni-/multivariate analysis OS (median overall: 52.0 (14.1–89.8) months)

	Univariate analysis		Multivariate analysis	
Parameter	Median lPFS (95% CI)	Significance	Hazard ratio (95% CI)	Significance
**Age**				
< 65 years	63.0 (14.2–112)	*p* = 0.828	-	-
≥ 65 years	31.8 (5.9–57.6)			
**Sex**				
Male	31.8 (12.0–51.6)	*p* = 0.623	-	-
Female	52.0 (9.7–94.2)			
**Histology**				
Adeno	28.8 (20.4–37.2)	*p* = 0.317	-	-
Squamous/other	52.0 (21.5–82.5)			
**Stage**				
IIIA	23.6 (22.0–25.2)	*p* = 0.079	-	-
IIIB	77.5 (11.4–143)			
IIIC	52.0 (0–108)			
**Therapy**				
CRT-IO	Not reached	*p* = 0.007	3.5 (1.2–10.6)	*p* = 0.023
CRT	25.2 (11.5–38.9)			
**Induction chemotherapy**				
Yes	52.0 (13.3–90.6)	*p* = 0.803	-	-
No	31.7 (14.1–89.8)			
**Cum. RT dose**				
< 60 Gy	23.0 (5.9–40.2)	*p* = 0.095	-	-
≥ 60 Gy	52.0 (19.1–84.8)			
**Residual MTV MS**				
< 4.3 ml	63.0 (42.9–83.2)	*p* = 0.003	3.9 (1.6–9.1)	*p* = 0.002
≥ 4.3 ml	23.0 (12.5–33.4)			
**SUV**_**max**_				
< 13.8	63.0 (41.3–84.7)	*p* = 0.016	2.3 (0.9–5.6)	*p* = 0.067
≥ 13.8	24.9 (17.7–32.2)			
**PTV**				
< 700 ml	63.0 (18.7–107)	*p* = 0.017	2.6 (1.2–6.1)	*p* = 0.026
≥ 700 ml	23.0 (15.7–30.4)			

To account for different, previously published cut-off values for rMTV (i.e., 1.0 ml and 25.0 ml rMTV), these values were also analyzed using univariate analysis comprising analogous results compared to the currently chose median split approach (please see Table 5).

**Table 5:** Uni-/multivariate analysis for further residual MTV cut-off values

	Median PFS (95% CI)	Hazard ratio PFS (95% CI)	Median LPFS (95% CI)	Hazard ratio LPFS (95% CI)	Median OS (95% CI)	Hazard ratio OS (95% CI)
**rMTV 1.0 ml**						
< 1.0 ml	29.3 (2.1–56.5)	-	49.9 (4.3–95.5)	3.4	63.0 (43.0–83.1)	3.1
≥ 1.0 ml	13.5 (8.2–18.7)		15.3 (11.4–19.1)	(1.5–7.7)	28.8 (19.9–37.7)	(1.3–7.6)
**Significance**	*p* = 0.105	-	*p* = 0.015	*p* = 0.003	*p* = 0.023	*p* = 0.013
**rMTV 25.0 ml**						
< 25.0 ml	19.7 (8.9–30.5)	3.3	49.9 (10.2–89.6)	4.2	63.0 (23.7–102)	5.0
≥ 25.0 ml	10.7 (6.2–15.2)	(1.6–6.9)	13.5 (7.5–19.5)	(1.9–9.1)	19.2 (11.2–27.2)	(2.1–12.1)
**Significance**	*p* = 0.020	*p* = 0.001	*p* = 0.004	*p* < 0.001	*p* = 0.009	*p* < 0.001

### Subgroup analyses—influence of rMTV in CRT/CRT-IO patients

In the overall group, significant influence of both the extent of rMTV and the application of CRT-IO in stage III NSCLC patients were described. Therefore, subgroups of patients receiving with CRT or CRT-IO were analyzed with regard to influence of rMTV on clinical outcome.

In the subgroup of patients receiving CRT only, the rMTV cut-off of 4.3 ml remained in strong association with PFS (median 33.5 vs. 8.6 months, *p* = 0.001). Also, smaller rMTV was associated with longer LPFS (median 49.9 vs. 10.1 months, *p* = 0.001) and OS (63.0 vs. 16.3 months, *p* = 0.001).

**Fig. 1:**
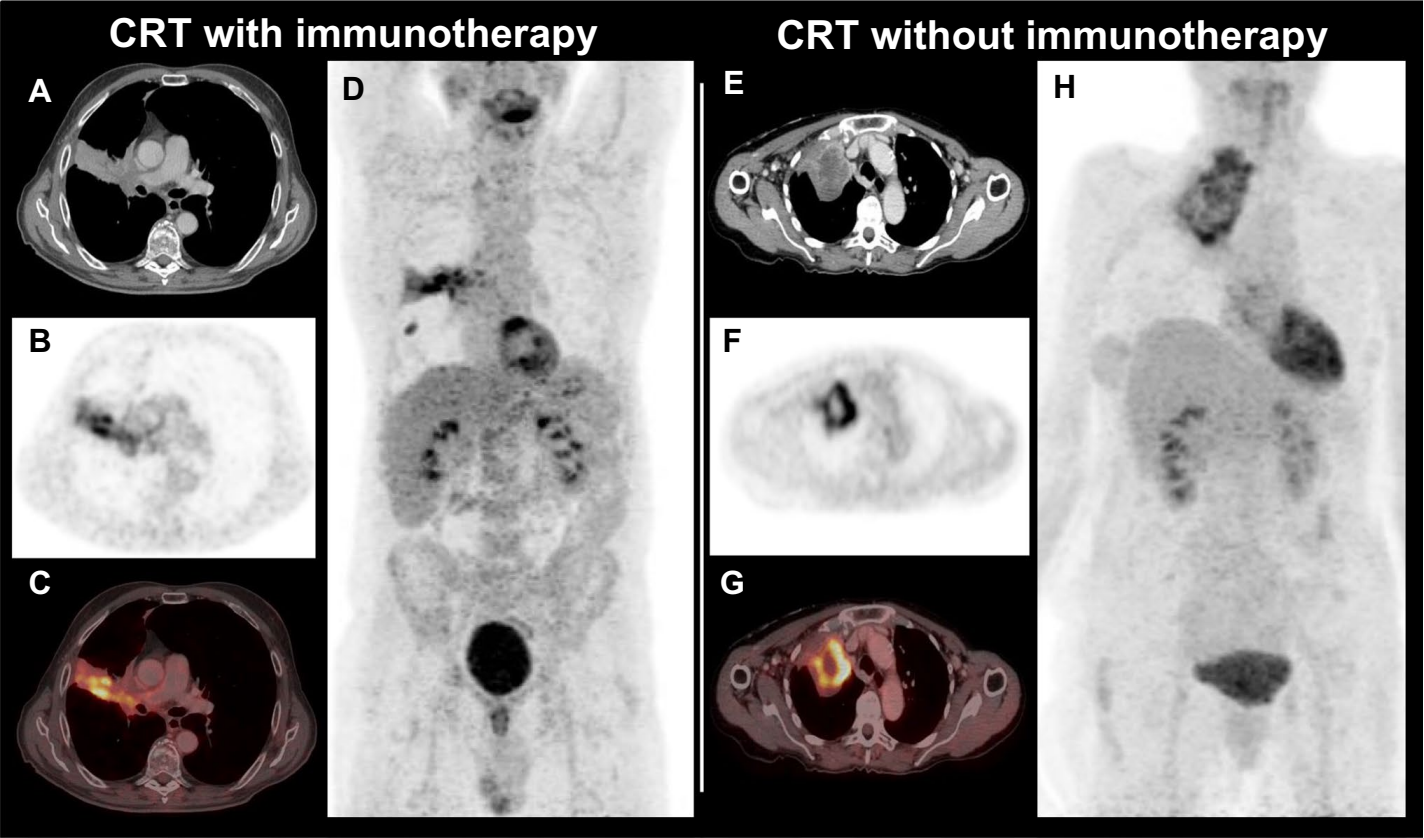
Left: NSCLC patient with CRT-IO and rMTV of 124.0 ml, but OS of at least 43.6 months (still during follow-up). Right: NSCLC patients with CRT only and rMTV of 116.0 ml, but comparably low OS of 8.0 months. A/E, contrast-enhanced CT; B/F, ^18^F-FDG PET; C/G, fused PET/CT; D/H, maximum intensity projections (MIP)

In contrast to the overall and CRT cohorts, no association of rMTV and clinical outcome was observed in the CRT-IO group; here, PFS (median 29.3 vs. 19.7, *p* = 0.909), LPFS (median not reached vs. 33.5 months, *p* = 0.291), and OS (median not reached in both groups, *p* = 0.720) were comparable between patients with small and larger rMTV (please see Table 6; for a patient example, please see Fig. 1).

**Table 6:** Residual MTV vs. CRT ± IO

Outcome parameter/residual MTV	Overall median (95% CI)	CRT-IO median (95% CI)	CRT median (95% CI)
**PFS**			
< 4.3 ml	29.3 (8.5–50.1)	29.3 (9.9–48.7)	33.5 (4.7–62.3)
≥ 4.3 ml	10.5 (6.7–14.3)	19.7 (n.a.)	8.6 (7.6–9.6)
Significance	*p* = 0.015	*p* = 0.909	*p* = 0.001
**LPFS**			
< 4.3 ml	49.9 (5.3–94.5)	Not reached	49.9 (0.0–106.3)
≥ 4.3 ml	13.5 (7.7–9.3)	33.5 (8.7–58.3)	10.1 (7.6–12.5)
Significance	*p* = 0.001	*p* = 0.291	*p* = 0.001
**OS**			
< 4.3 ml	63.0 (42.9–83.2)	Not reached	63.0 (42.2–83.8)
≥ 4.3 ml	23.0 (12.5–33.4)	Not reached	16.3 (9.7–22.8)
Significance	*p* = 0.003	*p* = 0.720	*p* = 0.001

*n.a.* not available.

## Discussion

The present study was designed to investigate a prognostic role of rMTV in inoperable stage III NSCLC patients treated with CRT and CRT-IO. All patients had completed primary multimodal treatment as well as post-treatment ^18^F-FDG PET/CT in the same institution.

In the overall outcome analysis, we found that a smaller rMTV after therapy completion and the subsequent application of ICI maintenance treatment were strongly associated with favorable outcome with significant hazards in the multivariate analyses. This was the case for PFS, locoregional control (i.e., LPFS), and OS. Of note, the size of the PTV was also a prognosticator for OS with contribution in the multivariate analysis without significant inter-correlation with rMTV. Other clinical parameter (e.g., TNM-stage) or image-derived parameters (e.g., SUV_max_) were not relevant as prognosticators in the present study.

Despite the association of CRT-IO and rMTV with favorable clinical outcome, we could demonstrate in the subsequent subgroup analyses that the extent of rMTV has a diverging role with special regard to the application of ICI maintenance therapy; in the subgroup of patients without ICI (i.e., CRT only), the extent of the rMTV was drastically associated with the clinical outcome. Vice versa, in the subgroup of CRT-IO patients, the rMTV was not associated with the clinical outcome (PFS, LRPFS, and OS) during the follow-up period.

Generally, rMTV was confirmed to be associated with patient survival. All tested rMTV values (1, 4, and 25 ml) have shown a correlation with LRPFS, PFS, and OS in the whole studied cohort. However, the principal finding was a different prognostic influence of rMTV in patients treated with and without ICI. Present results suggest that rMTV may have significantly lower impact on the locoregional tumor control as well as survival in patients treated with CRT-IO.

The role of initial and residual MTV and MTV changes after conventional CRT were previously described. Our group has shown that pre- and post-treatment MTV as well as at least 80% of their reduction after completion of CRT were significantly associated with overall survival [25, 26]. Both studies from Ohri et al. have also shown a significant correlation between pre-treatment total MTV and survival in locally advanced NSCLC after CRT. Additionally, a significant impact of “lesion_MTV” on the post-treatment local control was reported [34, 35]. Earlier, Machtay et al. in the cooperative ACRIN6668/RTOG0235 trial have revealed that a higher post-treatment tumor SUV is also associated with worse survival in locally advanced NSCLC after CRT [36].

Concerning CRT-IO as a new tri-modal treatment approach for inoperable stage III NSCLC, the data on PET/CT and metabolic parameters are very sparse. In the ground-breaking PACIFIC trial, an initial ^18^F-FDG-PET/CT was not obligatory because of a multi-national real-life study design and different general eligibility of the hybrid imaging. In addition, a blinded central-review radiological evaluation in the follow-up including duration of response was based on the conventional CT diagnostic (RECIST). Ohri et al. have recently published an exploratory retrospective single-center analysis evaluating an implementation of durvalumab maintenance after CRT. A survival benefit from durvalumab was found to be independent from initial MTV [37].

Ongoing translational and biomarker studies on CRT-IO will address a prognostic role of pre-and post-treatment ^18^F-FDG-PET/CT in lung cancer patients receiving CRT-IO. In the ESR 1814205 CRT-IO feasibility study in poor risk and/or elderly patients with stage III NSCLC, a ^18^F-FDG PET/CT is obligatory within 30 days for enrollment (NCT04441138). A phase II translational and biomarker DART study of the Norwegian group has incorporated a serial ^18^F-FDG-PET/CT imaging together with collection of tumor tissue as well as blood, urine, and stool samples [38]. For the ICI as a monotherapy in advanced NSCLC, several studies have already reported about a potential of metabolic parameters to predict post-treatment progression [39, 40]. A role of baseline MTV as a prognostic factor in metastatic NSCLC treated with nivolumab was described [41]. Additionally, a longitudinal ^18^F-FDG PET/CT analysis was also shown to have a potential to differentiate immune-dissociated response during ICI treatment [19, 42].

Within the current literature, what is a clinical implementation of our findings? Our study suggests a role of the ICI maintenance treatment after CRT as a “stabilizer” of the local–regional and distant tumor control as well as consequently LPFS and OS. A significant improvement of local tumor control with durvalumab after CRT was recently reported [43, 44]. This “stabilization” effect of ICI after CRT has a major impact on the significant improvement of patient survival in inoperable stage III NSCLC. Of note, in the light with current literature, we observed a significant association of the rMTV with clinical outcome in the CRT patients; however, this effect was no longer present in patients with subsequent ICI (i.e., CRT-IO group). This emphasizes the phenomenon that patients with large rMTV after CRT completion may build a special high-risk subgroup that significantly profit from ICI maintenance therapy despite extensive rMTV on PET-imaging, as no clinical outcome parameter in the CRT-IO group was associated with the extent of rMTV on PET—a drastic difference to patients without ICI.

Hence, the immunological interpretation of the rMTV after CRT is a phenomenon that must be addressed in future studies. A re-biopsy of the metabolic active region after CRT may be necessary to clarify the background of this phenomenon, and future biomarker studies need to consider this invasive procedure. Complementary, a serial investigation of circulating tumor DNA as a marker of molecular residual disease and longitudinal peripheral immunophenotyping may give information concerning interactions between local and systemic anti-tumor immune response. According to limitations of present study, a retrospective design and relatively low patient number must be mentioned. However, completion of all diagnostic and treatment procedures within a same tertiary cancer center as well as a comprehensive statistical analysis supports the present results.

## Conclusion

Overall, ICI maintenance treatment and rMTV are significantly associated with clinical outcome in inoperable stage III NSCLC after CRT. Despite its strong prognostic role in CRT alone patients, the extent of rMTV in the CRT-IO subgroup is not associated with outcome; hence, even patients with extensive rMTV after CRT completion significantly profit from ICI maintenance treatment.
